# Community health workers' mobility in China: Evidence from 2008 to 2017

**DOI:** 10.3389/fpubh.2022.947984

**Published:** 2022-08-23

**Authors:** Qi Zou, Xiaoqun He, Liang Zhang

**Affiliations:** ^1^College of Public Administration, Huazhong University of Science and Technology, Wuhan, China; ^2^Zhujiang Hospital, Southern Medical University, Guangzhou, China; ^3^School of Political Science and Public Administration, Wuhan University, Wuhan, China

**Keywords:** community health workers, mobility, retention, attraction, China

## Abstract

**Background:**

Community health workers (CHWs) are essential to improve the responsiveness of the national health system and the capacity of community health services. Since the implementation of the new program for health system reform in 2009, China has adopted an unprecedented policy to attract and retain CHWs to increase the quantity and quality of CHWs equitably. The policy's effects need to be further determined. The purpose of this study was to illustrate the temporal and spatial dynamics of CHWs' mobility in China from 2008 to 2017.

**Methods:**

This study used a multistage stratified sampling method to collect 14,094 CHW mobility records from 24 counties and 12 districts in six provinces in China for analysis. The data cover the period from 2008 to 2017. Descriptive statistics and chi-square test were adopted to analyse the distribution of mobile CHWs across different years and different subgroups from 2008 to 2017.

**Results:**

This study found that China's CHWs were in a state of net inflow from 2008 to 2017. The number of net inflows continued to increase from 2008 to 2011 and had a slight downward trend afterwards. CHW turnover occurred more in rural areas and amongst males, physicians, management and support staff, intermediate and senior professional titles, ≥30 years old group and contractual temporary employees. By contrast, the attraction and retention of CHWs are remarkable amongst nurses, pharmacists, imaging and laboratory technicians, junior title, junior college degree and above and <30 years old group.

**Conclusions:**

China has made great achievements with the policy targeting the attraction and retention of CHWs since the implementation of the new program for health system reform in 2009. However, CHWs in China are faced with the dilemma of an extremely high total mobility, the attraction of CHWs in rural areas is still insufficient, the professional structure of CHWs is unreasonable, and the attrition of high-quality health workers exists. China must pay attention to the fair allocation of urban and rural areas, dynamically adjust the recruitment structure of health technicians, improve the mechanism for attracting and retaining technicians with higher titles and guarantee the benefits of temporary employees.

## Introduction

Community health institutions (including urban community and rural township health services centers) are the most critical force in the primary health care system that provide community members with integrated and continuous health services in all aspects of medical care, public health, rehabilitation, immunization, health counseling and other aspects (1–4). As the main body of health service delivery in community health institutions, community health workers (CHWs) are critical to public access to accessible, equitable, affordable and high-quality primary health services, and their numbers and capacities are directly related to the performance related to sustainability of health system (5, 6). CHWs are the first point of contact between patients and the health system and undertake the vast majority of primary health services. Indeed, CHWs are the strongest backing to protect the health of all people. However, the shortage of CHWs in many areas around the globe, especially in poor, remote and rural areas, has brought a substantial negative impact on the protection of people's health (7).

CHWs can be divided into two categories according to community health service needs and the composition of the community general practitioner team: (1) professional CHWs, including physicians, nurses and health technicians (such as pharmacists, imaging and laboratory technicians) with medical practice certificates; (2) lay CHWs, namely, any non-medical professional workers involved in the provision of community health services, including technical staff without medical practice certificates (such as nutritionists, health trainers, rehabilitation therapists) and management and support staff (such as health counselors, health record managers, health service facilitators, administrators) (4–6, 8–11). China adopts the definition of CHWs in a broad sense to determine their composition, that is, CHWs refer to employees who work in community health institutions that play the main role in providing primary health services, including physicians, nurses, health technicians, other technical staff and management and support staff who have been on the job for more than half a year (12). In China, community health institutions are often welfare and non-profit institutions organized by the government. CHWs include two categories: permanent employees recruited by the government and temporary employees hired by community health institutions in the form of contracts.

CHW shortage has been a common phenomenon all over the world for a long time. The shortage occurs in low- and middle-income countries and regions in Africa, Asia and the Pacific, as well as in high-income countries, such as the United States, the United Kingdom and Switzerland (13, 14). CHW shortage is manifested as the outflow of human resources. For example, a 2012–2016 cohort study from Shanghai, China showed that community physicians from rural areas attrit (15). The United States faces the same predicament, with 16% of community physicians from Massachusetts report that they are less likely to stay at their workplace for 5 years (15). A 2009–2015 cohort study from Kenya showed that the incidence of attrition of CHWs reached 4.68% per year (2). The CHW shortage in developed and developing countries has not yet improved in recent years, and the turnover rate of CHWs remains relatively high.

The WHO has made several efforts and appeals to promote the level and equality of global CHW allocation. In 2010, the WHO put forward recommendations to increase the retention rate of CHWs in remote and rural areas through four aspects: education, compulsory measures, economic incentives and personal career support (4). In 2016, the WHO released the “Global Strategy on Human Resources for Health: Workforce 2030” (GSHRH), which determined the overall goal as “universal availability, accessibility, acceptability, coverage and quality of the health workforce through adequate investments to strengthen health systems and the implementation of effective policies at national, regional and global levels” (16). GSHRH regarded attracting and retaining high-quality health workers in the community as the main goal of strengthening the health system, improving performance and promoting health equity. The recommended strategies include but are not limited to the optimization of health workers' motivation, satisfaction, retention, equitable distribution and performance and the expansion of health workers' education (16).

The majority of countries have adopted corresponding policies to ensure the supply of primary health care providers. For example, measures of the US government are devoted to areas where health services are weak, such as providing special fund support for the internship of rural family physicians, enacting a policy of tuition-free medical students in rural areas and ensuring that the wage level of rural CHWs is not lower than the minimum income standard, and urban and rural counterpart assistance (17). The Health Service System in the United Kingdom is considered one of the best medical service systems in the world for the implementation of general practitioner system and the government's planning of community health services (17). Zhu found that the autonomy of institutions and fiscal measures perform an active role in the attraction and retention of rural CHWs in Cambodia, China and Vietnam (18).

As the most populous developing country in the world and the earliest center of the severe acute respiratory syndrome (SARS) and coronavirus disease 2019 (COVID-19) pandemics, China's health system is facing tremendous pressure, and the mobility of CHWs deserves much attention. China has attached great importance to strengthening the health system after suffering from the serious damage of the SARS epidemic in 2003. Consequently, China issued a new program for health system reform in 2009, proposing the construction of public health service system, medical service system, medical security system and medicine supply system that cover urban and rural residents to form a four-in-one basic health system (19, 20). This program is also the basis of the infrastructure of China's medical and health system today. The program attaches great importance to increasing the number of CHWs and encourages high-quality health personnel to work in the community.

China has adopted a series of strategies to attract and retain CHWs, such as medical education, financial incentives, professional career support and social security, to strengthen the allocation of CHWs (18). These measures help community health institutions to attract and retain outstanding health workers; thus, the quantity and quality of China's CHWs have steadily increased (15). China's efforts are highly consistent with the main goals and strategies of GSHRH, and China has taken a big step forward in realizing GSHRH's global milestones (by 2030). However, China has high CHWs turnover and insufficient recruitment of high-quality talents owing to problems, such as low income, professional development restrictions and lack of sense of accomplishment (20). CHWs in underdeveloped areas are even in a state of net loss (20). The attraction and retention of CHWs in China are facing huge challenges, threatening the accessibility and equity of primary health care services.

Although many studies analyzed the mobility of CHWs, they focused on individual occupations, such as physicians or nurses, and the research area is usually limited to remote rural areas (13–15). The lack of comparison between different occupations and regions in CHWs mobility makes the structural changes in CHWs and the differences between urban and rural areas due to CHWs mobility difficult to capture. Furthermore, few studies fully analyzed the prevalence and dynamic changes of China's CHWs. Instead, relevant studies on China focused on the turnover intention of CHWs (21–25). Since China implemented the new program for health system reform in 2009, public health concepts and lifestyles have continued to develop and change. The layout of community health service institutions has adjusted rapidly, and the level of CHW allocation has received the government's focus. Therefore, the overall situation and dynamic mobility of China's CHWs must be analyzed. With a true understanding of CHWs as basis, the effects of China's CHWs' attraction and retention policy should be evaluated to provide evidence for the talent attraction and retention policy.

This study focuses on the trend of CHW mobility in China from 2008 to 2017 and the difference in sociodemographic characteristics of CHWs and clarifies the reasons and mechanisms of the results to make up for the limitations of previous studies and understand the temporal and spatial dynamics of CHW mobility since China implemented the new program for health system reform. The findings have reference value for the resolution of CHW shortage in China and even in countries or regions at different development stages.

## Materials and methods

### Study design

Our study used a multistage stratified sampling method with unequal probability. Firstly, six provinces (municipalities) in the eastern (Shandong Province and Guangdong Province), central (Hubei Province and Henan Province) and western regions (Guizhou Province and Chongqing Municipality) of China were selected according to geographical distribution and economic level. Secondly, cities in the sample provinces were divided into high- and low-income cities according to their per capita GDP, and two prefecture-level cities were randomly selected. Chongqing City directly enters the next stage of sampling because it is a municipality directly under the central government (provincial administrative level). Therefore, 11 sample cities were obtained in this stage. Thirdly, one district and two counties/county-level cities (with high- and low-economic levels) were randomly selected in normal sample prefecture-level cities. In particular, Shenzhen City in Guangdong Province has no counties or county-level cities; hence, two districts were selected in Shenzhen City, and two more counties/county-level cities were selected from another prefecture-level city in Guangdong Province as a supplement. Two districts and four counties (with two high-economic level counties and two low-economic level counties) were randomly selected directly from Chongqing City. Thirty-six sample counties (districts and county-level cities) were obtained. Finally, a cluster sampling method was adopted to select all township health centers and community health services centers in the sample counties (districts and county-level cities). The per capita GDP level was classified according to the 2017 Statistical Yearbooks of the sample provinces and cities.

### Data collection

The data in our study were surveyed and collected by investigators in 24 counties and 12 districts. The data were collected through two main steps. Firstly, preliminary data were collected by the health administrative department. The information management staff downloaded the CHW mobility report data of all community health institutions in the jurisdiction in 2008–2017 from the China National Health Statistics Information Network Direct Reporting System. The report data include the demographic and socioeconomic characteristics, practice and job title, inflow/outflow time and employment type of mobile CHWs. Personal private information, including name, ID number and home address, were removed before the database was submitted to the investigators. Secondly, the investigators contacted the person in charge of the community health institutions to supplement and modify the database according to the personnel file data to ensure data integrity and accuracy. The investigators cleaned and aggregated the data to form a database of CHW mobility in China. Owing to the long-time span involved, some sample institutions had missing data in individual years; hence, institutions with a substantial amount of missing information in a given year were excluded. The number of institutions and mobile CHWs in each year are shown in Table 1. A total of 14,095 CHW mobility records were included in the analysis.

**Table 1:** Number of mobile CHWs in each institution in China from 2008 to 2017.

**Year**	**Number of institutions**	**Inflow**	**Outflow**	**Average inflow**	**Average outflow**	**Average net inflow**
2008	179	395	138	2.21	0.77	1.44
2009	207	544	171	2.63	0.83	1.80
2010	228	557	189	2.44	0.83	1.61
2011	250	868	296	3.47	1.18	2.29
2012	274	861	401	3.14	1.46	1.68
2013	322	1,083	378	3.36	1.17	2.19
2014	327	1,087	504	3.32	1.54	1.78
2015	359	1,183	685	3.30	1.91	1.39
2016	389	1,533	701	3.94	1.80	2.14
2017	395	1,622	898	4.11	2.27	1.83

### Data analysis

This study adopted descriptive statistics including frequency distribution, mean analysis, and statistical graphics to analyze the annual average number of inflowed, outflowed and net inflowed CHWs of the sample institutions, and their dynamic changing trend from 2008 to 2017 in China. Chi-square test was carried out to determine the distributions of background characteristics of mobile CHWs in different years, and the differences of the background characteristics between inflowed and outflowed CHWs.

The background characteristics were characterized according to the area of community health institutions (rural or urban). The gender of CHWs was classified as male and female. According to age, the CHWs were classified into <30 years old group, 30–39, 40–49, 50–60 and >60 years old age groups. The professional technical title of CHWs was classified as no title, junior title, intermediate title and senior title. According to the professional title system of health professionals in China, health professional technical title refers to the health practice certification obtained by health professionals who have completed the training and work in health institutions for a specified number of years and have passed the practice qualification examination. Junior title refers to the first practice qualification obtained by medical school graduates through the national practice qualification examination after completing the first-line practice. Intermediate title (such as attending physician, responsible master nurse and responsible master pharmacist) and senior title (such as chief physician, chief nurse and chief pharmacist) are obtained upon the completion of the health care work for the specified years of the corresponding profession on the premise of obtaining the previous professional technical title and passing government-designated examinations (26). The educational level of CHWs was classified as bachelor's degree and above, junior college, senior high school and junior high school and below. The professional practice of CHWs was classified as physicians (including practicing physicians, practicing assistant physicians and trainee physicians), registered nurses, pharmacists, imaging and laboratory technicians, other health technicians, other technicians and management and support staff. The employment type of CHWs was classified as permanent employees and contractual temporary employees.

Microsoft Excel 2016 was used to enter the questionnaire data and verify the entry results. The data were processed and analyzed by Statistical Package for the Social Sciences software (Version 23.0; IBM Corp.).

### Ethical considerations

This study was approved by the ethics committee of Tongji Medical College, Huazhong University of Science and Technology (IORG No: IORG0003571). Informed consent was obtained from the participants enrolled in this study, and the respondents were assured that their participation was voluntary and that they could withdraw from the study at any time.

## Results

### Dynamic analysis of the overall situation of CHWs' mobility in China from 2008 to 2017

The number of inflowed and outflowed CHWs from all sample institutions from 2008 to 2017 were 9,733 and 4,361, respectively. The former was 2.23 times higher than the latter. The specific data are shown in Table 1. The results showed that from 2008 to 2017, the annual average inflowed and outflowed CHWs from each institution had a rapid upward trend. The average number of inflowed CHWs per institution rose from 2.21 in 2008 to 4.11 in 2017, and the latter is 1.95 times higher than the former. The average annual number of inflowed CHWs per institution rose from 0.77 in 2008 to 2.27 in 2017, and the latter is 2.95 times higher than the former. The average annual net inflowed CHWs per institution rose from 1.44 in 2008 to 1.83 in 2017, and the latter is 1.27 times higher than the former. In general, the annual net inflowed CHWs for each institution remained between 1.44 and 2.29.

**Figure 1:**
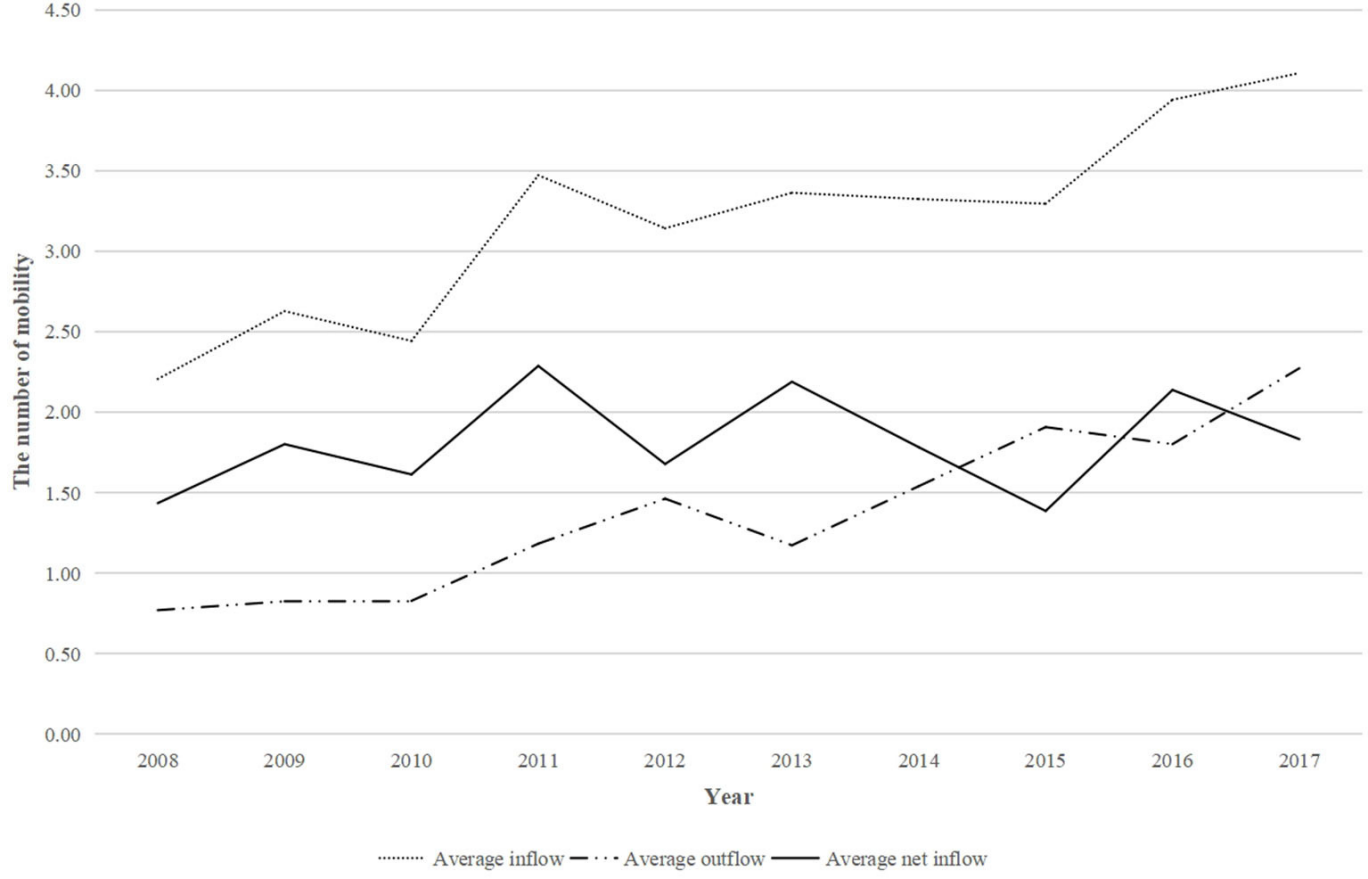
Trends in the number of mobile CHWs in each institution in China from 2008 to 2017.

Figure 1 shows that the average annual inflowed and outflowed CHWs from each institution in China remained unchanged in 2008–2010 and then the two grew steadily after 2010. Moreover, the average number of annual inflowed CHWs from 2008 to 2017 was significantly higher than that of outflowed CHWs, which means that the number of CHWs in China has grown during this period. The average annual net inflowed CHWs per institution had a relatively large increase (from 1.44 to 2.29) from 2008 to 2011, but the growth fluctuated from 2012 to 2017.

### Distribution of background characteristics of mobile CHWs by year in China from 2008 to 2017

#### Distribution of background characteristics of inflowed CHWs by year in China from 2008 to 2017

Table 2 indicates that the background characteristics of inflowed CHWs significantly vary from 2008 to 2017. Amongst the inflowed CHWs, the percentages of the rural group (χ^2^ = 56.91, *P* < 0.001), female group (χ^2^ = 30.68, *P* < 0.001), <39 years old group (χ^2^ = 63.12, *P* = 0.003), physicians and nurses (χ^2^ = 30.68, *P* < 0.001), junior title group (χ^2^ = 157.00, *P* < 0.001), junior college degree group (χ^2^ = 119.02, *P* < 0.001) and permanent employees (χ^2^ = 145.49, *P* < 0.001) were significantly higher than those of their counterparts. In terms of changing trends, the percentages of the urban group, female group, 40–49 years old age group, nurses, junior titles group, junior college degree group and contractual temporary employees continued to increase. Conversely, the percentages of the rural group, physicians, intermediate title group, 30–39 years old group and permanent employees continued to decline.

**Table 2:** Distribution of background characteristics of inflowed CHWs by year in China from 2008 to 2017.

**Background characteristics**	**2008**	**2009**	**2010**	**2011**	**2012**	**2013**	**2014**	**2015**	**2016**	**2017**	***χ^2^***	***P*-value**
**Area**												
Rural	343 (86.8)	456 (83.8)	421 (75.6)	718 (82.7)	675 (78.4)	799 (73.8)	847 (77.9)	910 (76.9)	1,225 (79.9)	1,254 (77.3)	56.91	<0.001
Urban	52 (13.2)	88 (16.2)	136 (24.4)	150 (17.3)	186 (21.6)	284 (26.2)	240 (22.1)	273 (23.1)	308 (20.1)	368 (22.7)		
Total	395 (100.0)	544 (100.0)	557 (100.0)	868 (100.0)	861 (100.0)	1,083 (100.0)	1,087 (100.0)	1,183 (100.0)	1,533 (100.0)	1,622 (100.0)		
**Gender**												
Male	147 (37.2)	207 (38.1)	176 (31.6)	259 (29.8)	262 (30.4)	357 (33.0)	302 (27.8)	340 (28.7)	476 (31.1)	496 (30.6)	30.68	<0.001
Female	248 (62.8)	337 (61.9)	381 (68.4)	609 (70.2)	599 (69.6)	726 (67.0)	785 (72.2)	843 (71.3)	1,057 (68.9)	1,126 (69.4)		
Total	395 (100.0)	544 (100.0)	557 (100.0)	868 (100.0)	861 (100.0)	1,083 (100.0)	1,087 (100.0)	1,183 (100.0)	1,533 (100.0)	1,622 (100.0)		
**Age**												
<30	254 (64.8)	374 (69.1)	384 (69.3)	544 (62.7)	563 (65.6)	730 (67.7)	747 (68.8)	790 (66.9)	1,037 (67.7)	1,042 (64.6)	63.12	0.003
30–39	103 (26.3)	121 (22.4)	124 (22.4)	213 (24.6)	186 (21.7)	219 (20.3)	217 (20)	246 (20.8)	311 (20.3)	376 (23.3)		
40–49	20 (5.1)	34 (6.3)	35 (6.3)	87 (10)	84 (9.8)	91 (8.4)	85 (7.8)	108 (9.2)	141 (9.2)	154 (9.5)		
50–59	12 (3.1)	10 (1.8)	8 (1.4)	19 (2.2)	17 (2)	31 (2.9)	23 (2.1)	22 (1.9)	19 (1.2)	27 (1.7)		
≥60	3 (0.8)	2 (0.4)	3 (0.5)	4 (0.5)	8 (0.9)	8 (0.7)	13 (1.2)	14 (1.2)	23 (1.5)	15 (0.9)		
Total	392 (100.0)	541 (100.0)	554 (100.0)	867 (100.0)	858 (100.0)	1,079 (100.0)	1,085 (100.0)	1,180 (100.0)	1,531 (100.0)	1,614 (100.0)		
**Professional practice**												
Physician	197 (53.4)	266 (51.4)	260 (49.7)	377 (46.6)	354 (43.2)	505 (49.4)	429 (42.5)	523 (46.4)	711 (48.5)	679 (44.3)	111.41	<0.001
Nurse	101 (27.4)	151 (29.2)	165 (31.5)	297 (36.7)	289 (35.2)	328 (32.1)	352 (34.9)	408 (36.2)	470 (32.1)	495 (32.3)		
Pharmacist	24 (6.5)	20 (3.9)	26 (5)	39 (4.8)	45 (5.5)	54 (5.3)	59 (5.8)	52 (4.6)	70 (4.8)	90 (5.9)		
Imaging and laboratory technician	10 (2.7)	19 (3.7)	24 (4.6)	33 (4.1)	60 (7.3)	54 (5.3)	75 (7.4)	65 (5.8)	106 (7.2)	109 (7.1)		
Other health technician	6 (1.6)	10 (1.9)	12 (2.3)	14 (1.7)	21 (2.6)	18 (1.8)	27 (2.7)	21 (1.9)	37 (2.5)	42 (2.7)		
Other technician	8 (2.2)	19 (3.7)	14 (2.7)	21 (2.6)	25 (3)	26 (2.5)	23 (2.3)	19 (1.7)	31 (2.1)	48 (3.1)		
Management and support staff	23 (6.2)	33 (6.4)	22 (4.2)	28 (3.5)	26 (3.2)	38 (3.7)	44 (4.4)	40 (3.5)	40 (2.7)	70 (4.6)		
Total	369 (100.0)	518 (100.0)	523 (100.0)	809 (100.0)	820 (100.0)	1,023 (100.0)	1,009 (100.0)	1,128 (100.0)	1,465 (100.0)	1,533 (100.0)		
**Professional technical title**												
Junior	260 (77.4)	377 (79.9)	390 (79.6)	627 (83.3)	637 (83.1)	824 (86.1)	798 (87.1)	917 (87.8)	1,182 (88.7)	1,173 (86.6)	157.00	<0.001
Intermediate	64 (19)	84 (17.8)	86 (17.6)	107 (14.2)	112 (14.6)	111 (11.6)	86 (9.4)	89 (8.5)	91 (6.8)	121 (8.9)		
Senior	6 (1.8)	9 (1.9)	12 (2.4)	13 (1.7)	11 (1.4)	17 (1.8)	11 (1.2)	16 (1.5)	30 (2.3)	27 (2)		
None	6 (1.8)	2 (0.4)	2 (0.4)	6 (0.8)	7 (0.9)	5 (0.5)	21 (2.3)	22 (2.1)	29 (2.2)	34 (2.5)		
Total	336 (100.0)	472 (100.0)	490 (100.0)	753 (100.0)	767 (100.0)	957 (100.0)	916 (100.0)	1,044 (100.0)	1,332 (100.0)	1,355 (100.0)		
**Education**												
Junior high school and below	8 (2.1)	10 (1.9)	7 (1.3)	12 (1.4)	8 (0.9)	11 (1)	8 (0.8)	7 (0.6)	9 (0.6)	6 (0.4)	119.02	<0.001
Senior high school	110 (28.3)	131 (24.3)	118 (21.3)	202 (23.4)	168 (19.9)	191 (17.9)	186 (17.4)	248 (21.2)	304 (19.9)	324 (20.3)		
Junior college	166 (42.7)	236 (43.7)	253 (45.8)	414 (48)	387 (45.8)	525 (49.1)	571 (53.6)	637 (54.4)	814 (53.3)	879 (55.1)		
Bachelor degree and above	105 (27.0)	163 (30.2)	175 (31.6)	234 (27.1)	282 (33.4)	342 (32)	301 (28.2)	278 (23.8)	399 (26.1)	385 (24.2)		
Total	389 (100.0)	540 (100.0)	553 (100.0)	862 (100.0)	845 (100.0)	1,069 (100.0)	1,066 (100.0)	1,170 (100.0)	1,526 (100.0)	1,594 (100.0)		
**Employment type**												
Contractual temporary	75 (20.1)	106 (20.1)	158 (29.3)	180 (21.5)	253 (30.5)	348 (33.6)	361 (34.4)	396 (35)	513 (35.6)	587 (37.9)	145.49	<0.001
Permanent	299 (79.9)	421 (79.9)	381 (70.7)	656 (78.5)	577 (69.5)	687 (66.4)	687 (65.6)	736 (65)	928 (64.4)	963 (62.1)		
Total	374 (100.0)	527 (100.0)	539 (100.0)	836 (100.0)	830 (100.0)	1,035 (100.0)	1,048 (100.0)	1,132 (100.0)	1,441 (100.0)	1,550 (100.0)		

#### Distribution of background characteristics of outflowed CHWs by year in China from 2008 to 2017

Table 3 shows that the background characteristics of outflowed CHWs considerably vary from 2008 to 2017. Amongst the outflowed CHWs, the percentages of the rural group (χ^2^ = 34.56, *P* < 0.001), < 39 years old group (χ^2^ = 101.42, *P* < 0.001), physicians (χ^2^ = 75.66, *P* = 0.028), junior title group (χ^2^ = 43.93, *P* = 0.021), junior college degree group (χ^2^ = 119.02, *P* < 0.001) and permanent employees (χ^2^ = 84.23, *P* < 0.001) were significantly higher than their counterparts. No significant difference in gender ratio was found (χ^2^ = 10.92, *P* = 0.281). In terms of changing trends, the percentages of the <30 years old group, nurses, junior and senior title groups, junior college and higher degree group and contractual temporary employees continued to increase. By contrast, the percentages of physicians, permanent employees, CHWs with intermediate titles and CHWs serving rural areas continued to decline.

**Table 3:** Distribution of background characteristics of outflowed CHWs by year in China from 2008 to 2017.

**Background characteristics**	**2008**	**2009**	**2010**	**2011**	**2012**	**2013**	**2014**	**2015**	**2016**	**2017**	***χ^2^***	***P*-value**
**Area**												
Rural	124 (89.9)	145 (84.8)	161 (85.2)	224 (75.7)	303 (75.6)	317 (83.9)	409 (81.2)	523 (76.4)	577 (82.3)	717 (79.8)	34.56	<0.001
Urban	14 (10.1)	26 (15.2)	28 (14.8)	72 (24.3)	98 (24.4)	61 (16.1)	95 (18.8)	162 (23.6)	124 (17.7)	181 (20.2)		
Total	138 (100.0)	171 (100.0)	189 (100.0)	296 (100.0)	401 (100.0)	378 (100.0)	504 (100.0)	685 (100.0)	701 (100.0)	898 (100.0)		
**Gender**												
Male	69 (50.0)	63 (36.8)	80 (42.3)	118 (39.9)	185 (46.1)	147 (38.9)	212 (42.1)	286 (41.8)	284 (40.5)	375 (41.8)	10.92	0.281
Female	69 (50.0)	108 (63.2)	109 (57.7)	178 (60.1)	216 (53.9)	231 (61.1)	292 (57.9)	399 (58.2)	417 (59.5)	523 (58.2)		
Total	138 (100.0)	171 (100.0)	189 (100.0)	296 (100.0)	401 (100.0)	378 (100.0)	504 (100.0)	685 (100.0)	701 (100.0)	898 (100.0)		
**Age**												
<30	25 (18.7)	40 (24.1)	51 (27.4)	103 (35)	98 (24.7)	114 (30.6)	169 (34)	195 (29)	218 (31.3)	277 (31.2)	101.42	<0.001
30–39	52 (38.8)	53 (31.9)	69 (37.1)	79 (26.9)	151 (38.1)	121 (32.4)	151 (30.4)	231 (34.3)	208 (29.8)	266 (30)		
40–49	12 (9)	19 (11.4)	22 (11.8)	45 (15.3)	68 (17.2)	56 (15)	55 (11.1)	129 (19.2)	119 (17.1)	168 (18.9)		
50–59	26 (19.4)	32 (19.3)	33 (17.7)	41 (13.9)	48 (12.1)	51 (13.7)	65 (13.1)	55 (8.2)	76 (10.9)	100 (11.3)		
≥60	19 (14.2)	22 (13.3)	11 (5.9)	26 (8.8)	31 (7.8)	31 (8.3)	57 (11.5)	63 (9.4)	76 (10.9)	76 (8.6)		
Total	134 (100.0)	166 (100.0)	186 (100.0)	294 (100.0)	396 (100.0)	373 (100.0)	497 (100.0)	673 (100.0)	697 (100.0)	887 (100.0)		
**Professional practice**												
physician	72 (57.1)	91 (54.5)	103 (57.9)	155 (54.4)	231 (59.1)	190 (54)	247 (51.4)	354 (54.3)	343 (51.9)	429 (50.9)	75.66	0.028
Nurse	27 (21.4)	44 (26.3)	42 (23.6)	87 (30.5)	95 (24.3)	95 (27)	120 (24.9)	158 (24.2)	190 (28.7)	266 (31.6)		
pharmacist	4 (3.2)	9 (5.4)	6 (3.4)	17 (6)	20 (5.1)	20 (5.7)	29 (6)	35 (5.4)	22 (3.3)	38 (4.5)		
Imaging and laboratory technician	2 (1.6)	5 (3)	6 (3.4)	6 (2.1)	15 (3.8)	9 (2.6)	28 (5.8)	30 (4.6)	25 (3.8)	24 (2.8)		
Other health technician	5 (4)	2 (1.2)	3 (1.7)	6 (2.1)	8 (2)	5 (1.4)	13 (2.7)	24 (3.7)	21 (3.2)	18 (2.1)		
Other technician	3 (2.4)	0 (0)	3 (1.7)	3 (1.1)	7 (1.8)	8 (2.3)	12 (2.5)	7 (1.1)	17 (2.6)	16 (1.9)		
Management and support staff	13 (10.3)	16 (9.6)	15 (8.4)	11 (3.9)	15 (3.8)	25 (7.1)	32 (6.7)	44 (6.7)	43 (6.5)	52 (6.2)		
Total	126 (100.0)	167 (100.0)	178 (100.0)	285 (100.0)	391 (100.0)	352 (100.0)	481 (100.0)	652 (100.0)	661 (100.0)	843 (100.0)		
**Professional technical title**												
Junior	73 (67)	100 (66.7)	122 (74.4)	198 (75)	265 (72.8)	232 (73.7)	337 (77.3)	441 (75.5)	464 (77.5)	556 (72.4)	43.93	0.021
Intermediate	36 (33)	45 (30)	36 (22)	52 (19.7)	86 (23.6)	68 (21.6)	84 (19.3)	122 (20.9)	105 (17.5)	171 (22.3)		
Senior	0 (0)	1 (0.7)	6 (3.7)	11 (4.2)	12 (3.3)	12 (3.8)	12 (2.8)	16 (2.7)	22 (3.7)	32 (4.2)		
None	0 (0)	4 (2.7)	0 (0)	3 (1.1)	1 (0.3)	3 (1)	3 (0.7)	5 (0.9)	8 (1.3)	9 (1.2)		
Total	109 (100)	150 (100)	164 (100)	264 (100)	364 (100)	315 (100)	436 (100)	584 (100)	599 (100)	768 (100)		
**Education**												
Junior high school and below	10 (7.8)	7 (4.2)	8 (4.3)	7 (2.4)	15 (3.9)	14 (3.8)	25 (5)	12 (1.8)	23 (3.3)	13 (1.5)	139.74	<0.001
Senior high school	73 (57)	76 (45.8)	76 (41.1)	129 (44.6)	165 (43)	126 (34.4)	184 (37)	217 (32.1)	243 (35.3)	246 (28.1)		
Junior college	38 (29.7)	68 (41)	78 (42.2)	111 (38.4)	147 (38.3)	158 (43.2)	210 (42.3)	333 (49.2)	303 (44)	414 (47.3)		
Bachelor degree and above	7 (5.5)	15 (9)	23 (12.4)	42 (14.5)	57 (14.8)	68 (18.6)	78 (15.7)	115 (17)	119 (17.3)	203 (23.2)		
Total	128 (100)	166 (100)	185 (100)	289 (100)	384 (100)	366 (100)	497 (100)	677 (100)	688 (100)	876 (100)		
**Employment type**												
Contractual temporary	8 (6.3)	18 (12.2)	29 (17.5)	69 (26.3)	70 (19.1)	61 (18.4)	121 (26.3)	153 (25)	182 (28.8)	254 (32.3)	84.23	<0.001
Permanent	118 (93.7)	130 (87.8)	137 (82.5)	193 (73.7)	297 (80.9)	270 (81.6)	339 (73.7)	460 (75)	450 (71.2)	532 (67.7)		
Total	126 (100)	148 (100)	166 (100)	262 (100)	367 (100)	331 (100)	460 (100)	613 (100)	632 (100)	786 (100)		

### Differences in the background characteristics between inflowed and outflowed CHWs

Chi-square test was adopted to analyze the differences in the background characteristics between China's inflowed and outflowed CHWs from 2008 to 2017. The analysis results are shown in Table 4. The results show that the area, gender, age, practice category, professional title, education and employment type have remarkable effects on CHW mobility.

**Table 4:** Differences in the socioeconomic characteristics of China's mobile CHWs from 2008 to 2017.

**Background characteristics**	**Inflowed CHWs (*n*, %)**	**Outflowed CHWs (*n*, %)**	**Total (*n*, %)**	**χ^2^**	***P*-value**
**Area**					
Rural	7,648 (78.58)	3,500 (80.26)	11,148 (79.10)	5.13	0.023
Urban	2,085 (21.42)	861 (19.74)	2,946 (20.90)		
Gender
Male	3,022 (31.05)	1,819 (41.71)	4,841 (34.35)	151.81	<0.001
Female	6,711 (68.95)	2,542 (58.29)	9,253 (65.65)		
**Age**					
< 30	6,465 (66.64)	1,290 (29.98)	7,755 (55.38)	2,230.00	<0.001
30–39	2,116 (21.81)	1,381 (32.09)	3,497 (24.97)		
40–49	839 (8.65)	693 (16.11)	1,532 (10.94)		
50–59	188 (1.94)	527 (12.25)	715 (5.11)		
≥60	93 (0.96)	412 (9.57)	505 (3.61)		
**Professional practice**					
Physician	4,301 (46.77)	2,215 (53.55)	6,516 (48.87)	136.30	<0.001
Nurse	3,056 (33.23)	1,124 (27.18)	4,180 (31.35)		
pharmacist	479 (5.21)	200 (4.84)	679 (5.09)		
Imaging and laboratory technician	555 (6.03)	150 (3.63)	705 (5.29)		
Other health technician	208 (2.26)	105 (2.54)	313 (2.35)		
Other technician	234 (2.54)	76 (1.84)	310 (2.33)		
Management and support staff	364 (3.96)	266 (6.43)	630 (4.73)		
**Professional technical title**					
Junior	7,185 (85.31)	2,788 (74.29)	9,973 (81.91)	257.40	<0.001
Intermediate	951 (11.29)	805 (21.45)	1,756 (14.42)		
Senior	152 (1.80)	124 (3.30)	276 (2.27)		
None	134 (1.59)	36 (0.96)	170 (1.40)		
**Education**					
Junior high school and below	86 (0.89)	134 (3.15)	220 (1.59)	538.92	<0.001
Senior high school	1,982 (20.62)	1,535 (36.07)	3,517 (25.36)		
Junior college	4,882 (50.78)	1,860 (43.70)	6,742 (48.61)		
Bachelor degree and above	2,664 (27.71)	727 (17.08)	3,391 (24.45)		
**Employment type**					
Contractual temporary	2,977 (31.97)	965 (24.80)	3,942 (29.86)	67.34	<0.001
Permanent	6,335 (68.03)	2,926 (75.20)	9,261 (70.14)		

In particular, the percentage of inflowed CHWs from rural areas was 78.58%, which was significantly lower than that in urban areas (21.42%, *P* < 0.001). The percentage of males amongst inflowed CHWs was 31.05%, which was significantly lower than that of females (68.95%, *P* = 0.023). The percentages of nurses, pharmacists, imaging and laboratory technicians amongst inflowed CHWs (33.23, 5.21 and 6.03%, respectively) were considerably higher than those amongst outflowed CHWs (27.18, 4.84 and 3.63, respectively). The percentages of physicians and management and support staff amongst inflowed CHWs (46.77 and 3.96%, respectively) were significantly lower than the those amongst outflowed CHWs (53.55 and 6.43%, respectively; *P* < 0.001). The junior title group (85.31%) of the inflowed CHWs was significantly higher than that of the outflowed CHWs (74.29%), whereas the intermediate and senior title groups of the inflowed CHWs were significantly lower than those of the outflowed CHWs (*P* < 0.001). The percentage of inflowed CHWs with junior college degree and above (78.49%) was significantly higher than the that of outflowed CHWs (60.78%), and the percentage of inflowed CHWs with high school education and below (21.51%) was significantly lower than that of the outflowed CHWs (39.22%, *P* < 0.001). The percentage of the <30 age group in inflowed CHWs (66.64%) was significantly higher than that in outflowed CHWs (29.98%), and the percentage of the ≥30 years old group in inflowed CHWs was significantly lower than that in outflowed CHWs (*P* < 0.001). The percentage of contractual temporary employees in inflowed CHWs (31.97%) was significantly higher than that in outflowed CHWs (24.80%, *P* < 0.001).

In summary, the mobile CHWs in rural areas and contractual temporary employees of China increased from 2008 to 2017. The inflow rate of nurses, pharmacists, imaging and laboratory technicians and CHWs with junior college degree and above in community health institutions was high. The outflow rate of males, physicians and management and support staff was high as well. Most of the inflowed CHWs had junior titles, and those with intermediate and senior titles were more likely to turnover. The age of inflowed CHWs was <30 years old, and those aged ≥ 30 years old were more likely to leave the community health institutions.

## Discussion

As a developing country with the largest population, China has huge health service needs. However, the lack of access, equity, affordability and quality of primary health services due to the shortage of CHWs is still the most important challenge faced by China's primary health services system. The need for a full range of integrated and continuous community health services means that strengthening CHW allocation is extremely important, and attention must be paid to the mobility of professional and lay CHWs to determine CHW attraction and retention strategies. Understanding the characteristics of CHWs mobility in China and the efforts made in attracting and retaining CHWs can provide reference for improving health workforce strategies in developing countries around the world.

Therefore, this study was based on 14,094 CHW mobility records from 24 counties and 12 districts of six provinces in eastern, central and western China between 2008 and 2017. The dynamic distribution of the number and socioeconomic characteristics of mobile CHWs in different years, as well as the differences in the socioeconomic characteristics between inflowed and outflowed CHWs, were analyzed in detail. This study intended to clarify the trends of CHW mobility before and after the implementation of the new program for health system reform in China in 2009. The results can reveal the laws and effects of CHW attraction and retention in China and provide evidence-based decision support for CHW attraction and retention. The main findings of this study are as follows.

China's CHWs maintained a stable net inflow state from 2008 to 2017, and the growth trend was particularly obvious from 2008 to 2011. The main reason is that the Chinese government has intensively implemented a series of strategies for attracting and retaining CHWs since the new program for health system reform was issued in 2009. The strategies include but are not limited to increasing CHWs' income through financial subsidies (27–29); the full implementation of CHWs on-the-job training project (27); encouraging medical school graduates to serve the community through tuition subsidies (27–29); order-oriented free training of medical students for the community (29, 30); CHWs' career promotion security (27, 28); special recruitment program for general practitioners (29); specifying the minimum number of general practitioners by region and population to ensure the accessibility and equity of general practitioners (31, 32) and special introduction projects for CHWs in poor, remote and rural areas (29, 33). The Chinese government has issued and implemented extremely powerful policies, systems, management mechanisms and financial and human security since 2009 to increase the number and equity of CHW allocation. These policies are the key driving force for the continuous increase in the number of CHWs in China from 2008 to 2017.

Compared with urban areas, rural areas are less attractive to CHWs, and this finding has been extensively discussed and verified (13, 25, 34). The main reason is the low service capacity and low utilization of community service institution in rural areas in China (35). The low income and limited career development of CHWs in these areas cause the problem of turnover. The rapid increase in urbanization rate, the low level of infrastructure construction and the lack of public service resources in rural areas are also important reasons for the resignation of CHWs in rural areas.

Male CHWs have a higher turnover rate than female CHWs, which is similar to the results of many previous studies (25, 36–38). The main reason is that, according to traditional Chinese culture, males take on more financial expenses in the family, such as housing purchases, car purchases and daily consumption expenditures. Therefore, male CHWs tend to seek higher income and are more willing to work in general hospitals for career development. However, the wages of Chinese community service agencies are relatively low, and the career development of CHWs is limited (20).

CHWs below 30 years old are more likely to be attracted and retained in community health institutions, which is contrary to the results of previous studies, especially those in other countries (36, 39). The main reason is that since 2009, China has introduced a large number of medical graduates to work in the community through targeted training, salary increase, career development and other incentive measures. These measures have led to a net inflow of young CHWs at the institutional level, which has reversed the aging trend of CHWs.

Although China's CHWs have been in a net inflow state from 2008 to 2017, the percentage of physicians has continued to decline, similar to the results of some previous studies (25, 35). The main reason is that the doctor-to-nurse ratio in China was too high in the past, and the number of nurses has a large gap. Therefore, since 2009, a large number of nurses have been recruited to improve the nursing capacity of the community. The results of this study also proved that the decrease in the percentage of inflowed physicians is due to the continuous increase in the percentage of inflowed nurses. However, the data show that the doctor-to-nurse ratios of China's community health service institutions were 1:1.28 in 2017 and 1:1.25 in 2018. The plan of the National Health Commission of China to reach a doctor-to-nurse ratio of 1:1.25 in 2020 was realized ahead of schedule (40). Therefore, the continuing decline in the percentage of physicians in the inflowed CHWs in China must be reversed in time. Besides, the management and support staff are more likely to be lost. The main reason is that the CHWs' attraction and retention policy in China's new program for health system reform focuses on health technicians. Community health institutions are increasingly encouraging health technicians to participate in management work, and health technicians are gradually taking part-time management and support jobs in China.

CHWs with higher titles are more likely to turnover. The main reason is that China's community health institutions deal with common and frequent diseases, which are simple and repetitive. Senior health personnel have difficulties of exerting a professional value in this situation. Additionally, higher-level hospitals in China have a “siphon effect” on senior health personnel by virtue of their superb medical and scientific research skills (23, 41).

CHWs with a high degree of education are more likely to be attracted and retained, which is contrary to the results of some previous studies, especially those in other countries (36). The main reason is similar to the state of net inflow of young CHWs in China. China's CHWs' attraction and retention policies include economic and non-economic incentives that are conducive to attract fresh medical graduates to work in the community.

Contractual temporary CHWs are more likely to attrit than permanent CHWs, similar to the results of previous studies (37, 42). The main reason is that permanent CHWs in China are hired by the government and their salaries are subsidized by the government. By contrast, contractual temporary CHWs are hired by community health institutions, and their salaries are paid by community health service agencies. The workload of temporary CHWs is the same as that of permanent CHWs, but their income and social security levels are lower than permanent employees. Moreover, the job security and stability of temporary employees are insufficient.

This study found that since China implemented the new program for health system reform in 2009, governments at all levels have taken unprecedented measures, and China has made tremendous progress in attracting and retaining CHWs. China's CHWs have been in a stable net inflow for a long time, the professional and technical structure tends to be reasonable, and the net inflow of young and highly educated CHWs is obvious. China's successful strategies include but are not limited to dedicated CHW education project, sound on-the-job training project, government-specific subsidies to raise CHWs' wages, provision of more permanent positions from the government, dedicated career development channels and a combination of economic and non-economic incentives. These huge experiments and their achievements in China hopefully provide a very good example for the improvement of CHWs' attraction and retention policies in many countries, especially in developing countries.

However, the mobility of CHWs in China exposes several problems, such as the decline in the percentage of inflowed CHWs from rural areas, the high percentage of physicians facing too-low risk and the high turnover rates of CHWs with higher titles and contractual temporary employees. Therefore, China's CHWs' attraction and retention policies could be further improved through the following measures: (1) enhance the recruitment and deployment of CHWs in rural areas; (2) adjust the percentage of CHWs in each category in a reasonable, timely and dynamic manner according to residents' health service demands; (3) design career development path and incentive measures to be more in line with CHWs' higher titles, improve the special review mechanism of CHWs' titles and make CHWs stay in the community through a combination of flexibility and rigidity measures; and (4) reduce the income and social security gap between temporary employees and permanent employees and ensure that the labor compensation of temporary employees is similar to that of permanent employees to increase their enthusiasm and reduce the turnover rate.

This study has some limitations that need to be addressed in further research. Firstly, the characteristic data between the flow of CHWs and on-duty CHWs cannot be compared and analyzed; thus, the analytic accuracy of the influencing factors of CHWs' mobility is insufficient. Secondly, in addition to personal factors and institutional factors, factors affecting CHWs' mobility include institutional factors, family factors and social factors. Further research on the effects of more comprehensive factors on CHWs' mobility is needed. Finally, the effect evaluation of China's large number of CHWs' attraction and retention policies requires further empirical analysis to improve the accuracy of policy effect analysis.

## Conclusions

The mobility of CHWs has a decisive effect on the number and quality of talents in community health institutions and the service capacity of community health institutions. This research shows that China's CHWs maintained a net inflow state in 2008–2017. The number of CHWs maintained a steady growth, the professional technology structure became more reasonable, and the trends of younger and higher education in CHWs were obvious. The results showed that China has made great achievements in the CHWs' attraction and retention policy since the implementation of the new program for health system reform in 2009. However, the results of CHWs' mobility in China from 2008 to 2017 showed that the effectiveness of the CHWs' attraction and retention policies declined. The attraction and retention in rural areas declined. Other problems arose, such as the percentage of physicians facing too-low risk and the high turnover rates of CHWs with higher titles and contractual temporary employees. The improvement of China's CHWs' attraction and retention policy needs to pay attention to the fair allocation of CHWs in urban and rural areas, dynamically adjust the recruitment structure of health technicians, improve the mechanism for attracting and retaining technicians with higher titles and guarantee the benefits of temporary employees.

## Data availability statement

The raw data supporting the conclusions of this article will be made available by the authors, without undue reservation.

## Ethics statement

The studies involving human participants were reviewed and approved by the Ethics Committee of Tongji Medical College, Huazhong University of Science and Technology (IORG No: IORG0003571). The patients/participants provided their written informed consent to participate in this study.

## Author contributions

QZ was involved in the conceptualization, methodology, data analysis, and writing of the original draft. XH was involved in the data curation, investigation, methodology, visualization, and writing of the original draft. LZ contributed to the conceptualization, methodology, and writing reviewing and editing. All authors revised the manuscript. All authors contributed to the article and approved the submitted version.

## Funding

This research was supported by the National Natural Science Foundation of China (Grant Number: 71734003). The funder had no role in the design of the study; the collection, analysis and interpretation of data or the writing of the manuscript.

## Conflict of interest

The authors declare that the research was conducted in the absence of any commercial or financial relationships that could be construed as a potential conflict of interest.

## Publisher's note

All claims expressed in this article are solely those of the authors and do not necessarily represent those of their affiliated organizations, or those of the publisher, the editors and the reviewers. Any product that may be evaluated in this article, or claim that may be made by its manufacturer, is not guaranteed or endorsed by the publisher.

## Abbreviations

CHWs: community health workers
SARS: the severe acute respiratory syndrome
COVID-19: coronavirus disease 2019
WHO: World Health Organization
GSHRH: Global Strategy on Human Resources for Health: Workforce 2030.
